# Efficacy and safety of transjugular intrahepatic portosystemic shunt versus endoscopic variceal ligation for variceal rebleeding: a systematic review and meta-analysis

**DOI:** 10.1097/MS9.0000000000003215

**Published:** 2025-04-02

**Authors:** Abdelaziz A. Awad, Alaa Ramadan, Abdelrahman M. Elettreby, Yousef Radwan Alnomani, Ahmed Hamdy Zabady, Esraa Abdelhafz, Yousef Ahmed El-Ayman, Mahmoud Ali, Merna R. Roshdy, Omar Abdelrahim, Moustafa S. Mohamed, Mohamed Elmasry, Naji Al-Bawah, Mohamed Fakhry

**Affiliations:** a Faculty of Medicine, Ai-Azhar University, Cairo, Egypt; b Faculty of Medicine, South Valley University, Qena, Egypt; c Faculty of Medicine, Mansoura University, Mansoura, Egypt; d Faculty of Medicine, Benha University, Benha, Egypt; e Department of microbiology, Faculty of Science, Damanhour University, Damanhour, Egypt; f Faculty of Pharmacy, Modern University for Technology & Information, Cairo, Egypt; g Faculty of Medicine, Lecturer, Department of General surgery, Zagazig University, Zagazig, Egypt; h Faculty of Medicine, Al-Azhar University, Damietta, Egypt; i Faculty of Medicine, Sohag University, Sohag, Egypt; j Theodore Bilharz Research Institute, Giza, Egypt; k Sednawi Hospital for Health Insurance, Cairo, Egypt; l Faculty of Medicine, Alexandria University, Alexandria, Egypt; m Faculty of Medicine, Sana’a University, Sana’a, Yemen; n Department of Hepatology and Gastroenterology and Infectious Diseases, Al-Azhar University, Assuit, Egypt

**Keywords:** cirrhosis, endoscopic variceal ligation, EVL, meta-analysis, TIPS, transjugular intrahepatic portosystemic shunt, variceal bleeding

## Abstract

**Background:**

Variceal bleeding is a significant cause of morbidity and mortality among patients with cirrhosis. While both transjugular intrahepatic portosystemic shunt (TIPS) and endoscopic variceal ligation (EVL) are utilized for variceal rebleeding prevention, their comparative efficacy and safety remain debated.

**Methods:**

A systematic review and meta-analysis were conducted to compare TIPS with EVL for variceal rebleeding prevention. A comprehensive search of electronic databases on PubMed, Embase, Scopus, and Web of Science identified 16 studies meeting inclusion criteria. Data on outcomes including gastrointestinal bleeding, variceal rebleeding, hepatic encephalopathy, treatment failure, and mortality were extracted and analyzed.

**Results:**

TIPS was associated with significantly lower rates of gastrointestinal bleeding (RR = −0.69, 95% CI [−0.92, −0.47], *P* < 0.001), variceal rebleeding (RR: −0.99, 95% CI [−1.2, −0.79], *P* < 0.001), and bleeding from banding ulcers (RR: −1.51, 95% CI [−2.75, −0.27], *P* = 0.02) compared to EVL. However, TIPS was linked to higher rates of hepatic encephalopathy (RR: 0.44, 95% CI [0.18, 0.71], *P* < 0.001) and treatment failure (RR: −1.29, 95% CI [−2.01, −0.57], *P* < 0.001). No significant differences were found in mortality, liver failure, hepatocellular carcinoma, or other clinical outcomes between the two interventions.

**Conclusion:**

TIPS demonstrates superiority over EVL in reducing variceal rebleeding and gastrointestinal bleeding. However, it is associated with higher rates of hepatic encephalopathy and treatment failure. Individualized treatment decisions should consider patient characteristics and treatment goals to optimize outcomes in variceal bleeding management. Further research is warranted to refine treatment strategies and minimize adverse events associated with both interventions.

**HIGHLIGHTS**

- TIPS significantly reduces variceal rebleeding and gastrointestinal bleeding compared to EVL.
- TIPS is associated with higher rates of hepatic encephalopathy and treatment failure.
- No significant differences in mortality or other major outcomes between TIPS and EVL.
- Careful patient selection is key to optimizing outcomes and minimizing complications.
- Long-term follow-up and individualized treatment approaches are essential for effective management of variceal bleeding.

## Introduction

Variceal bleeding is a leading cause of morbidity and death. Between 2 years of diagnosis, varices are expected to develop in 30–40% of cirrhotic patients, and variceal hemorrhage to occur in 30% of these patients. Despite advancements in medical and endoscopic therapy, the death rate from variceal hemorrhage remains significant, ranging from 20% to 50%.^[1,2]^ The primary underlying cause of variceal bleeding is portal hypertension, resulting from liver cirrhosis or, less commonly, non-cirrhotic portal hypertension. Portal hypertension leads to blood flow diversion into collateral vessels, including esophageal varices, which lack the normal structural support of vascular walls, making them susceptible to rupture and subsequent hemorrhage.^[3]^

To avoid variceal rebleeding, two primary therapeutic techniques are frequently utilized: endoscopic variceal ligation (EVL) and transjugular intrahepatic portosystemic shunt (TIPS). TIPS lowers portal pressure and stops variceal hemorrhage by establishing a shunt between the hepatic vein and the portal vein. Conversely, EVL involves variceal obliteration through the closure of esophageal varices with an endoscope. There is constant discussion about the relative safety and effectiveness of TIPS and EVL, despite the fact that both are useful in reducing variceal rebleeding. In order to optimize patient outcomes and inform clinical decision-making, it is imperative to comprehend the relative advantages of different therapies.^[4,5]^

It has been demonstrated that TIPS works very well to lower portal pressure and stop variceal rebleeding. In terms of rebleeding rates and overall survival, TIPS has been shown to be superior to medical therapy alone in several clinical trials^[6,7]^. On the other hand, TIPS has several disadvantages, including as the possibility of hepatic encephalopathy, difficulties during the procedure, and the requirement for ongoing monitoring and upkeep. In contrast to TIPS, EVL is a less intrusive technique that carries a lesser risk of hepatic encephalopathy. The effectiveness of EVL in reducing variceal rebleeding has been shown in several trials, with results that are similar to TIPS in terms of overall survival and rebleeding rates. However, there is a chance of problems such stricture formation and esophageal ulcers, and EVL may need more than one session to completely eradicate the variceal.^[8,9]^ There is a need for a thorough assessment of the safety and effectiveness of TIPS and EVL in preventing variceal rebleeding, given their disadvantages. In patients with a history of variceal bleeding, how does TIPS compare to EVL in preventing variceal rebleeding and improving survival outcomes? This study aims to provide a comprehensive evaluation of the safety and efficacy of TIPS versus EVL to inform clinical decision-making and optimize patient management.

## Methods

### Search strategy

A systematic literature search will be conducted using electronic databases including PubMed, Scopus, Embase, and Web of Science. The search strategy will utilize a combination of keywords and Medical Subject Headings (MeSH) terms related to “TIPS,” “EVL,” “variceal bleeding,” and “cirrhosis,” The search will be limited to studies published in English, from inception until 20 January 2024. The search strategy and limitations of each database are shown in Table 1S, http://links.lww.com/MS9/A785. Additionally, reference lists of relevant articles and review papers will be manually screened to identify any additional studies missed by the electronic search. This study was registered in the International Prospective Register of Systematic Reviews (PROSPERO) under ID: CRD42024503872. The study has been reported in line with AMSTAR (Assessing the methodological quality of systematic reviews) Guidelines.

### Study selection

Two independent reviewers will screen the titles and abstracts of identified studies to determine their eligibility for inclusion. Any disagreement was resolved by a third senior author (M.F.). Full-text articles will be retrieved for potentially relevant studies, and eligibility will be assessed based on predefined inclusion and exclusion criteria. Inclusion criteria will encompass RCTs comparing the efficacy and safety of TIPS versus EVL for the prevention of variceal rebleeding in patients with variceal bleeding. All cases involved cirrhosis, and we explicitly state that no instances of pre-portal hypertension were included in our analysis. Studies must report outcomes such as variceal rebleeding rates, mortality, hepatic encephalopathy, procedural complications, and quality of life.

Studies were excluded if they: included patients with active or acute variceal bleeding (AVB), as our focus was on long-term prevention of rebleeding in patients who had already stabilized; used bare or non-dedicated stents for TIPS, given our aim to evaluate outcomes specifically with dedicated covered stents; involved study designs, populations, or interventions that did not allow for consistent comparisons within our target parameters; or involved pediatric populations.

### Data extraction

Data extraction will be performed independently by two reviewers using a standardized data extraction form. Extracted data will include study characteristics (e.g., study design, sample size, follow-up duration), patient demographics (e.g., age, sex, etiology of cirrhosis), intervention details (e.g., TIPS technique, EVL procedure), and outcomes of interest (e.g., variceal rebleeding rates, mortality, hepatic encephalopathy, complications). Any discrepancies between reviewers will be resolved through consensus or consultation with a third reviewer.

### Definitions

Variceal rebleeding is any episode of gastrointestinal bleeding that is determined to be caused by esophageal or gastric varices after first therapy. The majority of the included studies graded problems using the Clavien-Dindo classification system, which is generally acknowledged and provides for standardized reporting of postoperative complications.

### Quality assessment

The methodological quality of included studies will be assessed independently by two reviewers using appropriate tools for RCTs (e.g., Cochrane Risk of Bias Tool).^[10]^ Studies will be evaluated based on criteria such as randomization, allocation concealment, blinding, completeness of follow-up, and handling of missing data. The Cochrane Risk of Bias Tool examined studies’ adherence to major methodological principles and assigned them a “good” or “poor” quality rating. A study was deemed “good quality” if it demonstrated minimal risk in crucial areas such as randomization, allocation concealment, blinding, comprehensive follow-up, and proper treatment of missing data. In contrast, studies were considered “poor quality” if they had a high or uncertain risk of bias in any of these crucial dimensions. Discrepancies in quality assessment will be resolved through discussion or involvement of a third reviewer.

### Data synthesis and analysis

In our meta-analysis, we focused on predefined complications that are clinically relevant to the treatment of variceal bleeding. Specifically, we aimed to analyze the following complications: Variceal rebleeding, Hepatic encephalopathy, Ascites, Hepatorenal syndrome, Treatment failure Mortality, Liver failure, Hepatocellular carcinoma, Gastrointestinal bleeding, and Procedural complications (as graded by the Clavien-Dindo classification)

A meta-analysis was conducted to quantitatively synthesize the results of included studies using appropriate statistical methods. Pooled estimates of treatment effects were calculated using random-effects or fixed-effects models, depending on the level of heterogeneity, which was assessed using the *I*^2^ statistic. Heterogeneity was classified as low (*I*^2^ < 25%), moderate (*I*^2^ = 25%-50%), or substantial (*I*^2^ > 50%), in line with standard guidelines. Where necessary, transformation methods were applied to standardize outcome measures across studies, especially for continuous variables, to ensure consistency in pooled estimates. Publication bias was evaluated using funnel plots and Egger’s test. All analyses were performed using STATA software, with two-sided significance testing and a significance level set at *P* < 0.05.

## Results

**Figure 1.**
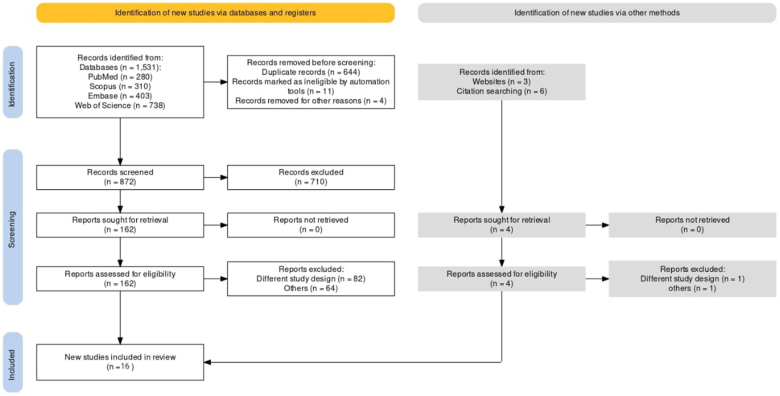
PRISMA flow diagram of the selection process.

A total of 1531 unique citations were screened through the literature search. After title and abstract screening, only 50 studies were eligible, and following further assessment by full text, 16 studies were included in this systematic review and meta-analysis. The PRISMA flowchart is shown in Fig. 1.

### Characteristics of included studies

A total of 16 studies were included in the systematic review and meta-analysis. The studies were conducted across various geographical regions, most common are Chine, Spain and Germany, and included diverse patient populations with cirrhotic variceal bleeding. The sample sizes ranged from 46 to 129 participants. The interventions compared were TIPS and EVL for the prevention of variceal rebleeding. The duration of follow-up varied among studies, ranging from 6 months to 5 years, with the primary outcomes being rebleeding rates, mortality, and adverse events related to the interventions (Table 1).

**Table 1:** Summary of the included studies

Study ID	Country	Number of patients in each group		Age (years) Mean (SD)		Gender (male) *N*. (%)		Child-Pugh-class (*n*%)						Child-Pugh-Score		Ascites (%)		Alcoholic sclerosis (%)		Viral sclerosis (%)		Covered or non-covered stents	Portosystemic pressure gradient	Embolization
								Class A		Class B		Class C		Mean/SD		TIPS	Endoscopic	TIPS	Endoscopic	TIPS	Endoscopic	TIPS	Post-TIPS placement	TIPS
		TIPS	Endoscopic	TIPS	Endoscopic	TIPS	Endoscopic	TIPS	Endoscopic	TIPS	Endoscopic	TIPS	Endoscopic	TIPS	Endoscopic									
Lv 2017^[11]^	China	84	45	50.7 ± 11.6	50.9 ± 10.4	53 (63%)	34 (76%)	–	–	17 (20%)	10 (22%)	19 (23%)	10 (22%)	8 ± 1.52	8 ± 1.53	–	7 (16%)	2 (2%)	4 (9%)	65 (78%)	34 (85%)	Covered	8 · 3 ± 2 · 4 mm Hg	Five patients
García-Pagán 2010^[11]^	Spain	32	31	52 ± 10	49 ± 6	21 (65.63%)	23 (74.2%)	–	–	16 (50%)	16 (51.6%)	16 (50%)	15 (48.4%)	9.3 ± 1.8	9.5 ± 1.8	19 (59.4%)	18 (58.1%)	22 (68.75%)	20 (64.5%)	4 (12.5%)	5 (16.1%)	Covered	Less that 12 mmHg	–
Gulberg2002^[12]^	Germany	28	26	57 ± 2	56 ± 2	20 (71.4%)	19 (73.1%)	11 (39.3%)	10 (38.5%)	15 (53.6%)	12 (46.2%)	2 (7.1%)	4 (15.4%)	–	–	–	–	22 (78.6%)	23 (88.5%)	3 (10.7%)	3 (11.5%)	Non-covered	–	Non
Chen 2022^[13]^	China	52	54	57.31 ± 1.25	53.72 ± 1.27	42 (79.25%)	46 (86.79%)	2 (3.85)	5 (9.26)	36 (69.23)	37 (68.52)	14 (26.92)	12 (22.22)	8.16 ± 2.12	7.87 ± 2.63	39 (75.00)	37 (68.51)	2 (3.85)	3 (5.56)	43 (82.7)	46 (85.2%)	Covered	Less than 12 mmHg	Four patients
Holster 2016^[14]^	Netherlands	37	35	56 ± 29.31	51.67 ± 31.7	18 (66)	23 (49)	13 (35)	13 (37)	19 (51)	18 (51)	5 (14)	4 (11)	7.5 6 2.0	7.3 6 1.9	15 (41)	13 (37)	13 (35)	18 (51)	7 (19)	1 (3)	Covered	4.4 6 ± 2.1 mmHg	8 patients
Jalan 1997^[15]^	Scotland	31	27	55.17 ± 9.5	59.9 ± 8.6	21 (67.74)	16 (59.25)	2 (6.5)	5 (18.5)	14 (45.2)	9 (33.33)	15 (48.4)	13 (48.1)	9.2 ± 2.3	9.1 ± 2.6	28 (90.2%)	21 (77.7%)	26 (83.8)	21 (77.77)	2 (6.5)	1 (3.7)	Non-covered	Less than 12 mmHg	Non
Lo 2007^[16]^	Taiwan	35	37	55 ± 11	52 ± 2	25 (80.6)	28 (75.7)	9 (25.7)	12 (32.4)	20 (57.1)	19 (51.4)	6 (17.14)	6 (17.21)	7.8 ± 1.8	7.6 ± 1.7	–	–	4 (11.4)	8 (21.6)	30 (85.71)	26 (70.2)	Non-covered	7.5 ± 3.5 mmHg	Non
Luo 2015^[17]^	China	37	36	50.78 ± 13.61	49.53 ± 14.02	19 (51.35)	24 (66.66)	–	–	25 (67.56)	24 (66.66)	12 (32.43)	12 (33.33)	8.76 ± 1.70	8.89 ± 1.77	–	–	2 (5.4)	4 (11.11)	30 (81.1)	26 (72.22)	Covered	10.4 ± 6 3.1 mmHg	21 patients.
Lv 2017^[18]^	China	24	25	52.33 ± 12.6	46.67 ± 14.15	13 (54)	16 (64)	9 (38)	10 (40)	13 (54)	14 (56)	2 (8)	1 (4)	–	–	3 (13)	2 (8)	1 (4)	0 (0)	21 (87)	22 (88)	Covered	8.7 ± 4.4 mm Hg	41 patients
Merli 1998^[19]^	Italy	38	73	60.5 ± 8.5	58.2 ± 10.7	31 (70.1)	27 (71.1)	13 (34.2)	13 (30.2)	20 (52.6)	25 (58.2)	5 (13.2)	5 (11.6)	–	–	–	–	6 (15.8)	15 (34.9)	27 (71.0)	19 (44.2)	Non-covered	10.6 ± 0.7 mm Hg	Non
Cabrea 1996^[20]^	Spain	31	32	55.67 ± 9.14	55.87 ± 12.5	20 (64.52)	23 (71.875)	14 (45.2)	14 (43.75)	13 (41.9)	16 (50)	4 (12.9)	2 (6.25)	7.1 ± 1.59	7.22 ± 1.75	10 (32.25)	11 (34.4)	20 (64.5)	23 (72%)	–	–	Non-covered	10.5 ± 2.8 mmHg	Non
Pomier-Layrargues 2000^[21]^	Canada	41	39	52.9 ± 13.3	54.3 ± 10.9	29 (70.73)	27 (69.23)	–	–	–	–	–	–	9.6 ± 1.6	9.8 ± 1.6	–	–	25 (60.97)	24 (61.53)	4 (9.7)	5 (12.8)	Non-covered	Below 12 mm Hg	Non
Sanyal 1997^[22]^	USA	41	39	48 ± 8	52 ± 6	26 (63.41)	27 (69.23)	7 (17.1)	6 (15.4)	13 (31.7)	15 (38.5)	21 (51.2)	18 (469.15)	–	–	14 (34.14)	12 (30.7)	16 (39)	17 (43.59)	18 (43.9)	18 (46.15)	Non-covered	11 ± 2.5 mm Hg	Non
Sauer 1997^[23]^	Germany	42	41	52.8 ± 9.5	60.2 ± 12.6	27 (64.28)	21 (51.2)	15 (35.7)	123 (29.26)	18 (42.85)	18 (43.9)	9 (21.4)	11 (26.8)	7.76 ± 2.29	8.26 ± 2.46	–	–	25 (59.5)	26 (63.41)	13 (30.95)	10 (24.39)	Non-covered	10-15 mmHg	Non
Sauer 2002^[24]^	Germany	43	42	53.5 ± 11.8	55.1 ± 12.5	27 (62.8)	23 (54.76)	15 (34.88)	10 (23.81)	16 (37.21)	19 (45.23)	12 (27.91)	13 (30.95)	7.9 ± 2.1	8.2 ± 2.0	15 (34.88)	19 (45.23)	29 (67.44)	24 (57.14)	9 (20.9)	12 (28.57)	Non-covered	12.1 ± 2.1 mmHg	Non
Narahara 2001^[25]^	Japan	38	40	51.3 ± 1.6	54.5 ± 1.6	32 (84.21%)	30 (75%)	–	–	–	–	–	–	6.8 ± 0.3	7.4 ± 0.3	10 (26.31)	14 (35)	9 (23.68)	17 (42.5)	24 (63.15)	20 (50)	Non-covered	9.4 ± 0.6 mmHg	Non

### Risk of bias assessments

**Figure 2.**
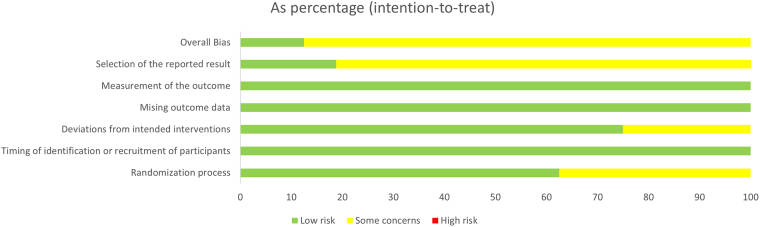
Risk of bias and summary of the included studies.

The risk of bias was evaluated for each included study using appropriate tools such as the Cochrane Risk of Bias Tool for RCTs, the assessment criteria included random sequence generation, allocation concealment, blinding of participants and personnel, incomplete outcome data, selective reporting, and other sources of bias. Overall, most RCTs demonstrated some concerns risk of bias, with adequate randomization and allocation concealment methods. However, blinding of participants and personnel was often not feasible due to the nature of the interventions. (Fig. 2)

### Outcomes

#### Bleeding-related outcomes

**Figure 3.**
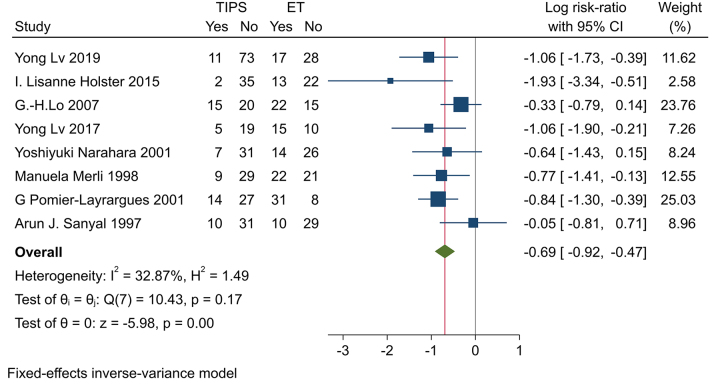
Forest Plot of the GI bleeding.

**Figure 4.**
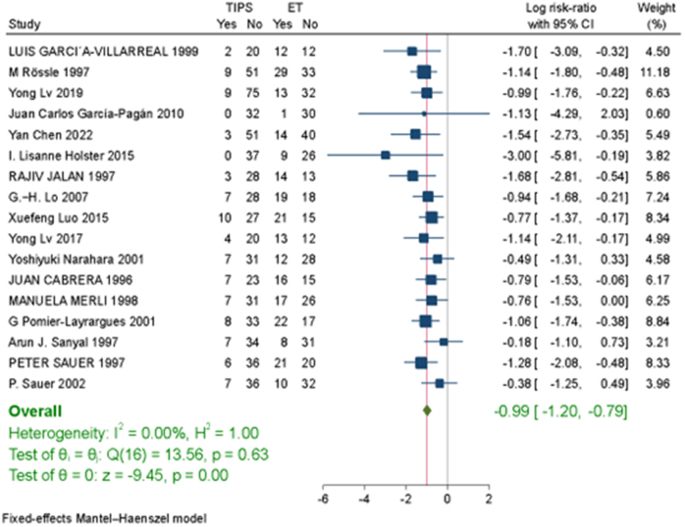
Forest Plot of the variceal rebleeding.

TIPS was associated with a significantly lower risk of GI bleeding compared to ES (RR = −0.69, 95% CI [−0.92, −0.47], *P* < 0.001; Fig. 3). TIPS demonstrated a significantly lower risk of variceal rebleeding (RR = −0.99, 95% CI [−1.2, −0.79], *P* < 0.001; Fig. 4). TIPS had a significantly reduced risk of bleeding from banding ulcers (RR = −1.51, 95% CI [−2.75, −0.27], *P* = 0.02; Figure. 1S. http://links.lww.com/MS9/A782). No significant difference was observed between TIPS and ES in the risk of bleeding from peptic ulcers (RR = −0.06, 95% CI [−0.95, 0.84], *P* = 0.9; Figure. 2S. http://links.lww.com/MS9/A782)

#### Risk of hepatic encephalopathy and ascites

**Figure 5.**
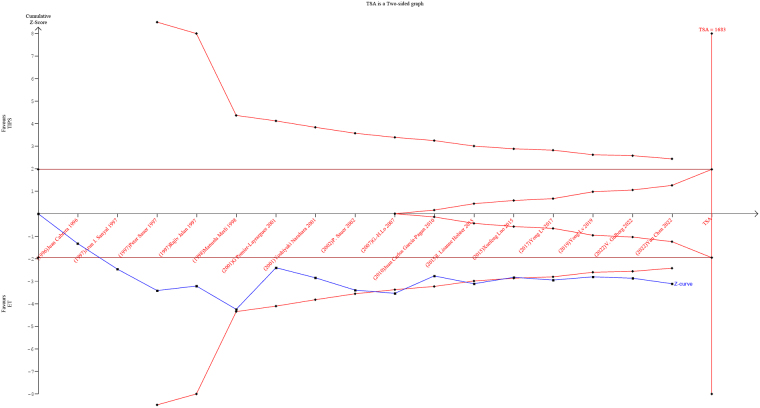
Trial sequential analysis of the hepatic encephalopathy.

TIPS was significantly associated with a higher risk of HE (RR = 0.44, 95% CI [0.18, 0.71], *P* < 0.001; Figure. 4S. http://links.lww.com/MS9/A782). Patients receiving TIPS had a significantly lower probability of developing ascites (RR = −1.06, 95% CI [−1.49, −0.62], *P* < 0.001; Figure. 5S. http://links.lww.com/MS9/A782).

#### Other liver-related outcomes

TIPS significantly reduced the risk of hepatorenal syndrome (RR = −1.04, 95% CI [−1.81, −0.27], *P* = 0.01; Figure 7S. http://links.lww.com/MS9/A782). No significant difference in liver failure (RR = 0.47, 95% CI [−0.2, 1.15], *P* = 0.17; Figure 9S. http://links.lww.com/MS9/A782) or HCC (RR = 0.59, 95% CI [−0.77, 1.95], *P* = 0.4; Figure 10S. http://links.lww.com/MS9/A782) was observed between TIPS and ES.

#### Additional complications and treatment success

TIPS was associated with a lower risk of dysphagia (RR = −1.7, 95% CI [−2.88, −0.53], *P* < 0.001; Figure 13S. http://links.lww.com/MS9/A782). Patients receiving TIPS had a reduced risk of peritonitis compared to ES (RR = −1.32, 95% CI [−2.35, −0.28], *P* = 0.01; Figure 14S. http://links.lww.com/MS9/A782). TIPS was associated with a significantly lower rate of treatment failure (RR = −1.29, 95% CI [−2.01, −0.57], *P* < 0.001; Figure 8S. http://links.lww.com/MS9/A782).

#### Mortality and other non-significant outcome

Mortality: There was no significant difference in mortality between TIPS and ES (RR = −0.1, 95% CI [−0.37, 0.17], *P* = 0.47; Figure. 4S. http://links.lww.com/MS9/A782). No differences were found in bleeding from ulcers, DVT, fatigue, fever, gastropathy, itching, need for transplantation, sepsis, or hydrothorax (Figures 11S–21S. http://links.lww.com/MS9/A782).

#### Heterogeneity and sensitivity analyses

Outcomes with homogeneous studies (*I*^2^ < 25%) included GI bleeding (*I*^2^ = 24.81%), variceal rebleeding (*I*^2^ = 0%), and bleeding from banding and peptic ulcers (*I*^2^ = 0% and *I*^2^ = 14.4%). Higher heterogeneity was noted in studies on treatment failure (*I*^2^ = 55.88%), mortality (*I*^2^ = 51.6%), fever (*I*^2^ = 73.12%), and hepatic encephalopathy (HE) (*I*^2^ = 43.59%). These studies underwent leave-one-out sensitivity analyses to assess robustness, with details shown in Figures 24S–27S. http://links.lww.com/MS9/A782.

Galbraith Plot: Used to identify studies with potential outliers, with one study outside the 95% CI in mortality but no outliers in treatment failure, fever, or HE analyses (Figure 28S. http://links.lww.com/MS9/A782).

#### Rehospitalization and hospitalization rates

Rebleeding Index, Hospitalization, and Rehospitalization Rates: These outcomes showed high heterogeneity (*I*^2^ = 96.54% for rebleeding index, *I*^2^ = 85.37% for hospitalization, and *I*^2^ = 98.57% for rehospitalization), with sensitivity analyses suggesting limited influence of individual studies (Figures 29S–32S. http://links.lww.com/MS9/A782).

Trial sequential analysis revealed that the evidence that TIPS decreases variceal rebleeding and increases the risk of HE is sufficient and conclusive and no more studies are needed (Fig. 5).

### Meta-regression

Neither Child-Pugh score, presence of alcoholic cirrhosis nor presence of hepatitic cirrhosis were found to influence the probability of variceal rebleeding (B-coefficient = −0.125 with 95% CI [−0.398, 0.148], SE = 0.139, *P* = 0.368), (B-coefficient = −0.004 with 95% CI [−0.021, 0.012], SE = 0.008, *P* = 0.61), and (B-coefficient = 0.003 with 95% CI [−0.01, 0.016], SE = 0.007, *P* = 0.64) respectively Figures 33S. http://links.lww.com/MS9/A782. However, the Child-Pugh score was a significant predictor of the occurrence of hepatic encephalopathy (B-coefficient = −0.426 with 95% CI [−0.692, −0.159], SE = 0.136, *P* = 0.002) Figure 34S. http://links.lww.com/MS9/A782. Still, neither presence of alcoholic cirrhosis nor the presence of hepatitic cirrhosis was found to predict HE (B-coefficient = 0.004 with 95% CI [−0.019, 0.274], SE = 0.012, *P* = 0.707), and (B-coefficient = −0.004 with 95% CI [−0.016, 0.015], SE = 0.008, *P* = 0.958) respectively Figures 35S. http://links.lww.com/MS9/A782.

### Publication bias

We used funnel plot to detect potential publication bias. Funnel plot for studies assessing mortality shows asymmetry which means possible publication bias. The trim and fill method found that two studies were needed to achieve stability Figures 36S. http://links.lww.com/MS9/A782. Funnel plot for studies assessing variceal rebleeding shows asymmetry, trim and fill method found that four studies were needed to achieve stability Figures 37S. http://links.lww.com/MS9/A782. Funnel plot for studies assessing HE shows asymmetry, trim and fill method found that seven studies were needed to achieve stability Figures 38S. http://links.lww.com/MS9/A782. Funnel plot for studies assessing sepsis shows asymmetry, trim and fill method found that one study was needed to achieve stability Figures 39S. http://links.lww.com/MS9/A782. Funnel plot for studies assessing treatment failure shows asymmetry Figure 40S. http://links.lww.com/MS9/A784.

### GRADE assessment

According to the GRADE system, the strength of evidence was high for variceal rebleeding, hepatic encephalopathy, peritonitis, GI bleeding, bleeding from banding ulcer, ascites and dysphagia; moderate for treatment failure, hepatocellular carcinoma, liver transplantation, gastropathy, fatigue, and bleeding from peptic ulcer; low for sepsis and hydrothorax; and very low for death, DVT, fever, ulcer, and itching (Table 2).

**Table 2:** GRADE assessment

Certainty assessment							No. of patients		Effect			
No. of studies	Study Design	Risk of bias	Inconsistency	Indirectness	Imprecision	Other considerations	TIPS	Endoscopic therapy	Relative (95% CI)	Absolute (95% CI)	Certainty	Importance
**Variceal rebleeding (assessed with: active bleeding or stigmata of thrombus on varices at endoscopy)**												
17	Randomized trials	Not serious	Not serious	Not serious	Not serious	Publication bias strongly suspected strong association^a^	96/689 (13.9%)	251/651 (38.6%)	**RR 0.100** (0.063 to 0.160)	**347 fewer per 1000** (from 361 fewer to 324 fewer)	⨁⨁⨁⨁ High	CRITICAL
**Hepatic encephalopathy (assessed with: altered consciousness and elevated arterial ammonia levels)**												
18	Randomized trials	Not serious	Not serious	Not serious	Not serious	Publication bias strongly suspected strong association^a^	224/717 (31.2%)	133/677 (19.6%)	**RR 2.75** (1.51 to 5.13)	**344 more per 1000** (from 100 more to 811 more)	⨁⨁⨁⨁ High	CRITICAL
**Peritonitis**												
7	Randomized trials	Not serious	Not serious	Not serious	Not serious	None	2/297 (0.7%)	12/254 (4.7%)	**RR 0.0480** (0.0045 to 0.5200)	**45 fewer per 1000** (from 47 fewer to 23 fewer)	⨁⨁⨁⨁ High	CRITICAL
**GI bleeding (assessed with: hematemesis or melena)**												
9	Randomized trials	Not serious	Not serious	Not serious	Not serious	None	88/398 (22.1%)	177/365 (48.5%)	**RR 0.18** (0.11 to 0.29)	**398 fewer per 1000** (from 432 fewer to 344 fewer)	⨁⨁⨁⨁ High	CRITICAL
**Sepsis (assessed with: blood culture)**												
10	Randomized trials	Not serious	Not serious	Not serious	Serious^b^	Publication bias strongly suspected^a^	34/420 (8.1%)	29/377 (7.7%)	**RR 1.20** (0.42 to 3.39)	**15 more per 1000** (from 45 fewer to 184 more)	⨁⨁◯◯ Low	CRITICAL
**Death (assessed with: vital signs monitoring)**												
18	Randomized trials	Not serious	Serious^c^	Not serious	Serious^b^	Publication bias strongly suspected^a^	173/717 (24.1%)	193/632 (30.5%)	**RR 0.79** (0.43 to 1.48)	**64 fewer per 1000** (from 174 fewer to 147 more)	⨁◯◯◯ Very low
**Treatment failure (assessed with: any uncontrolled rebleeding occurring anytime following both treatments or treatment switch)**												
10	Randomized trials	Not serious	Serious^c^	Not serious	Not serious	Publication bias strongly suspected strong association^a^	31/446 (7.0%)	93/412 (22.6%)	**RR 0.0510** (0.0098 to 0.2690)	**214 fewer per 1000 (from 224 fewer to 165 fewer)**	⨁⨁⨁◯ Moderate	CRITICAL
**Bleeding from peptic ulcer**												
6	Randomized trials	Not serious	Not serious	Not serious	Serious^b^	None	7/254 (2.8%)	7/214 (3.3%)	**RR 0.87** (0.11 to 6.92)	**4 fewer per 1000** (from 29 fewer to 194 more)	⨁⨁⨁◯ Moderate	CRITICAL
**Bleeding from banding ulcer**												
5	Randomized trials	Not serious	Not serious	Serious^d^	Not serious	Strong association	1/231 (0.4%)	10/190 (5.3%)	**RR 0.0300** (0.0018 to 0.5400)	**51 fewer per 1000** (from 53 fewer to 24 fewer)	⨁⨁⨁⨁ High	CRITICAL
**Ascites (assessed with: ultrasound ascites score)**												
5	Randomized trials	Not serious	Not serious	Serious^d^	Not serious	Strong association	25/231 (10.8%)	53/190 (27.9%)	**RR 0.087** (0.032 to 0.239)	**255 fewer per 1000** (from 270 fewer to 212 fewer)	⨁⨁⨁⨁ High	CRITICAL
**DVT**												
3	Randomized trials	Serious^e^,^f^	Not serious	Serious^d^	Very serious^b^	None	4/162 (2.5%)	3/124 (2.4%)	**RR 1.290** (0.056 to 29.510)	**7 more per 1000** (from 23 fewer to 690 more)	⨁◯◯◯ Very low	IMPORTANT
**Hepatocellular carcinoma**												
3	Randomized trials	Not serious	Not serious	Not serious	Very serious^b^	Strong association	7/146 (4.8%)	3/110 (2.7%)	**RR 3.89** (0.17 to 89.13)	**79 more per 1000** (from 23 fewer to 1000 more)	⨁⨁⨁◯ Moderate	IMPORTANT
**Liver transplantation**												
6	Randomized trials	Not serious	Not serious	Not serious	Serious^b^	None	17/261 (6.5%)	10/217 (4.6%)	**RR 2.51** (0.48 to 13.18)	**70 more per 1000** (from 24 fewer to 561 more)	⨁⨁⨁◯ Moderate	IMPORTANT
**Hepatorenal syndrome**												
5	Randomized trials	Not serious	Not serious	Not serious	Not serious	None	8/231 (3.5%)	19/190 (10.0%)	**RR 0.09** (0.02 to 0.54)	**91 fewer per 1000** (from 98 fewer to 46 fewer)	⨁⨁⨁⨁ High	IMPORTANT
**Liver failure**												
5	Randomized trials	Not serious	Not serious	Serious^d^,^g^	Serious^b^	None	19/188 (10.1%)	12/191 (6.3%)	**RR 2.95** (0.63 to 14.13)	**123 more per 1000** (from 23 fewer to 825 more)	⨁⨁◯◯ Low
**Gastropathy**												
7	Randomized trials	Not serious	Not serious	Not serious	Serious^b^	None	4/291 (1.4%)	7/252 (2.8%)	**RR 0.295** (0.026 to 3.467)	**20 fewer per 1000** (from 27 fewer to 69 more)	⨁⨁⨁◯ Moderate	IMPORTANT
**Dysphagia**												
6	Randomized trials	Not serious	Not serious	Not serious	Not serious	None	1/281 (0.4%)	15/245 (6.1%)	**RR 0.0199** (0.0013 to 0.2951)	**60 fewer per 1000** (from 61 fewer to 43 fewer)	⨁⨁⨁⨁ High	IMPORTANT
**Fatigue**												
2	Randomized trials	Not serious	Not serious	Not serious	Serious^b^	None	5/108 (4.6%)	5/70 (7.1%)	**RR 0.398** (0.028 to 5.623)	**43 fewer per 1000** (from 69 fewer to 330 more)	⨁⨁⨁◯ Moderate	NOT IMPORTANT
**Fever**												
3	Randomized trials	Not serious	Serious^c^	Not serious	Very serious^b^	None	14/168 (8.3%)	4/132 (3.0%)	**RR 4.169** (0.008 to 2238.720)	**96 more per 1000** (from 30 fewer to 1000 more)	⨁◯◯◯ Very low	NOT IMPORTANT
**Hydrothorax**												
2	Randomized trials	Not serious	Not serious	Not serious	Very serious^b^	None	2/108 (1.9%)	2/70 (2.9%)	**RR 0.417** (0.006 to 28.184)	**17 fewer per 1000** (from 28 fewer to 777 more)	⨁⨁◯◯ Low	NOT IMPORTANT
**Ulcer**												
2	Randomized trials	Serious^e^,^f^	Not serious	Serious^d^	Very serious^b^	None	7/138 (5.1%)	4/99 (4.0%)	**RR 1.51** (0.09 to 25.12)	**21 more per 1000** (from 37 fewer to 975 more)	⨁◯◯◯ Very low	NOT IMPORTANT
**Itching**												
2	Randomized trials	Not serious	Not serious	Not serious	Extremely serious^b^	None	3/121 (2.5%)	1/80 (1.3%)	**RR 3.09** (0.03 to 288.40)	**26 more per 1000** (from 12 fewer to 1000 more)	⨁◯◯◯ Very low	NOT IMPORTANT

CI: confidence interval; RR: risk ratio

^a^ funnel plot showing asymmetry

^b^ 95% CI is wide and includes a potential for important harm or benefit by crossing the null effect line

^c^ heterogeneity >50% and reduced extent of overlap of confidence intervals

^d^ population is restricted to HCC patients

^e^ deviation from the intended interventions (addition of beta blockers to the endoscopic group)

^f^ selection of the reported results

^g^ cyanoacrylate injection is used as a comparator.

## Discussion

This systematic review and meta-analysis included 18 studies comparing TIPS with EVL for variceal rebleeding prevention. TIPS was associated with significantly lower rates of gastrointestinal bleeding, variceal rebleeding, and bleeding from banding ulcers compared to EVL. However, TIPS was linked to higher rates of hepatic encephalopathy and treatment failure, along with lower rates of ascites and hepatorenal syndrome. No significant differences were found in mortality, liver failure, hepatocellular carcinoma, itching, need for liver transplantation, dysphagia, peritonitis, deep vein thrombosis, fatigue, fever, gastropathy, ulcers, sepsis, or hydrothorax between the two interventions. A huge number of clinical studies and meta-analyses show that TIPS surgery is not superior to endoscopic therapy in terms of improved survival time. This is the primary reason for using TIPS as a rescue option if established therapy methods have failed. Although the rescue TIPS operation is efficient in controlling acute bleeding, the postoperative one-year survival rate is approximately 27%-55%.^[26-29]^ The summary of the previous meta-analyses are showed in Table 3.

**Table 3:** Summary of the previous meta-analyses

Comparison	Our Study	Berengy et al. 2024^[30]^	Zhu et al. 2024^[31]^
Literature search	From inception until 20 January 2024	From inception to September 2023	From inception until May 18, 2023
Registration on PROSPERO	CRD42024503872	Nonregistered	CRD42023439185
Number of included studies	16	5	5
Quality assessment	ROB 2	ROB 2 (only summary)	ROB 2
GRADE assessment	Yes	No	No
Trial sequential analysis	Yes	No	No
Key Findings	TIPS significantly reduces rates of gastrointestinal bleeding (RR = −0.69, 95% CI [−0.92, −0.47], *P* < 0.001), variceal rebleeding (RR = −0.99, 95% CI [−1.2, −0.79], *P* < 0.001), and bleeding from banding ulcers (RR = −1.51, 95% CI [−2.75, −0.27], *P* = 0.02) compared to EVL. However, it is associated with higher rates of hepatic encephalopathy (RR = 0.44, 95% CI [0.18, 0.71], *P* < 0.001) and treatment failure (RR = −1.29, 95% CI [−2.01, −0.57], *P* < 0.001). No significant differences were observed in mortality or other major clinical outcomes.	TIPS significantly reduced the incidence of variceal bleeding (VB) (RR, 0.34; *P* < .0001) and mortality from acute gastrointestinal bleeding (RR, 0.37; *P* = .05). Although there was no significant difference in overall mortality or liver failure mortality between the groups, TIPS demonstrated a higher probability of remaining free from VB in the first 2 years post-procedure, indicating its therapeutic advantage over EBL plus propranolol.	TIPS significantly reduced the rate of variceal rebleeding compared to endoscopic therapy (ET) plus nonselective beta-blockers (NSBBs) in patients with cirrhosis and portal vein thrombosis (PVT) (OR = 0.19, 95% CI = 0.11–0.35, *P* < 0.05, *I*^2^ = 0%). Additionally, TIPS demonstrated a higher rate of portal vein recanalization (OR = 7.92, 95% CI = 3.04–20.67, *P* < 0.05, *I*^2^ = 0%). However, there was no significant difference between TIPS and ET + NSBBs regarding hepatic encephalopathy or mortality.

The observed lower rates of variceal rebleeding and gastrointestinal bleeding with TIPS align with previous evidence suggesting the superiority of TIPS in reducing portal pressure and preventing variceal hemorrhage.^[32,33]^ The creation of a portosystemic shunt through TIPS effectively decompresses the portal system, thereby reducing the risk of variceal rupture and subsequent bleeding. Similarly, the decreased incidence of bleeding from banding ulcers with TIPS may be attributed to the more comprehensive hemodynamic effects of this intervention compared to EVL, which primarily targets variceal obliteration.^[33,34]^ However, the higher incidence of hepatic encephalopathy associated with TIPS warrants careful consideration. Hepatic encephalopathy is a serious complication of liver disease, characterized by cognitive impairment and altered mental status, which can significantly impact patients’ quality of life and overall prognosis. The increased risk of hepatic encephalopathy with TIPS may be attributed to factors such as portosystemic shunting leading to worsened ammonia detoxification and alterations in cerebral perfusion.^[35]^ Despite advancements in TIPS techniques aimed at minimizing hepatic encephalopathy risk, this complication remains a concern and underscores the importance of patient selection and careful post-procedural management. Hepatic encephalopathy, in our included studies, was not increased in the early-TIPS group compared to EVL therapy, which is consistent with a prior meta-analysis and the AASLD guideline that recognizes no difference between the two therapies.^[36]^

Furthermore, the higher rates of treatment failure observed with TIPS raise questions regarding the long-term effectiveness and durability of this intervention in preventing variceal rebleeding. Treatment failure may encompass various factors, including shunt dysfunction, recurrence of varices, or development of new complications requiring intervention. This highlights the need for close monitoring of patients undergoing TIPS and consideration of adjunctive therapies or repeat interventions as needed to maintain therapeutic efficacy. Interestingly, no significant differences were found between TIPS and EVL in terms of mortality, liver failure, hepatocellular carcinoma, and several other clinical outcomes. This suggests that while TIPS may offer advantages in terms of reducing variceal rebleeding, it does not confer a clear survival benefit or mitigate other liver-related complications compared to EVL. The lack of significant differences in these outcomes underscores the importance of individualized treatment decisions based on patient characteristics, disease severity, and treatment goals.^[30,31]^

### Strengths and limitations

The systematic literature search conducted in multiple electronic databases ensures that relevant studies comparing TIPS and EVL for variceal rebleeding prevention were identified, the inclusion of RCTs and rigorous quality assessment using appropriate tools enhances the reliability and validity of the findings, The inclusion of 18 studies with diverse patient populations and interventions provides a substantial dataset for the meta-analysis, enhancing the generalizability of the results. However, some limitations could be found, some outcomes exhibited significant heterogeneity across studies, which may limit the interpretation of pooled estimates. However, subgroup and sensitivity analyses were performed to address this issue, while efforts were made to assess the risk of bias in included studies, the presence of potential biases, such as lack of blinding in certain studies, may affect the overall quality of evidence and asymmetric funnel plots and evidence of publication bias in some outcomes suggest the possibility of selective reporting or publication of studies with positive results.

### Research recommendations

Future research should focus on long-term follow-up studies to assess the durability of treatment effects, including variceal rebleeding rates, hepatic encephalopathy, and overall survival, Further comparative effectiveness studies are needed to evaluate the relative efficacy and safety of TIPS and EVL in specific patient subgroups based on disease severity, etiology of cirrhosis, and other clinical factors and cost-effectiveness analyses comparing TIPS and EVL in terms of healthcare resource utilization, procedural costs, and long-term management should be conducted to inform healthcare decision-making.

## Conclusion

In conclusion, this systematic review and meta-analysis provide valuable insights into the comparative effectiveness and safety of TIPS versus EVL for variceal rebleeding prevention in patients with cirrhosis. While TIPS demonstrates superiority in reducing variceal rebleeding and gastrointestinal bleeding compared to EVL, it is associated with higher rates of hepatic encephalopathy and treatment failure. Individualized treatment decisions should consider the trade-offs between efficacy and safety of each intervention, taking into account patient preferences, disease characteristics, and resource availability. Further research is warranted to address remaining uncertainties and optimize the management of variceal bleeding in cirrhotic patients.

## Data Availability

No new datasets were generated or analyzed in the preparation of this manuscript.

## Research registration unique identifying number (UIN)

ID: CRD42024503872.
